# Brassinosteroids as promoters of seedling growth and antioxidant activity under heavy metal zinc stress in mung bean (*Vigna radiata L*.)

**DOI:** 10.3389/fmicb.2023.1259103

**Published:** 2023-10-05

**Authors:** Naresh Kumar, Vikas Sharma, Gurpreet Kaur, Charu Lata, Hemant Dasila, Kahkashan Perveen, Faheema Khan, Vijay K. Gupta, Mehrun Nisha Khanam

**Affiliations:** ^1^Department of Chemistry and Biochemistry, Eternal University, Rajgarh, India; ^2^Department of Biochemistry, Kurukshetra University, Kurukshetra, India; ^3^ICAR-National Dairy Research Institute, Karnal, India; ^4^ICAR-Central Soil Salinity Research Institute, Karnal, India; ^5^ICAR- Indian Institute of Wheat and Barley Research, RRS, Shimla, India; ^6^Department of Microbiology, Eternal University, Rajgarh, India; ^7^Department of Botany and Microbiology, College of Science, King Saud University, Riyadh, Saudi Arabia; ^8^School of Biological Sciences, College of Natural Sciences, Seoul National University, Seoul, Republic of Korea

**Keywords:** brassinosteroids, EBL, mung bean, antioxidative enzymes, zinc, heavy metal

## Abstract

The escalation of harmful pollutants, including heavy metals, due to industrialization and urbanization has become a global concern. To mitigate the negative impacts of heavy metal stress on germination and early plant development, growth regulators have been employed. This study aimed to evaluate the response of mung bean (*Vigna radiata L.*) to zinc stress in the presence of brassinosteroids, focusing on seedling growth and antioxidant potential. Mung bean seedlings were treated with three concentrations of 24-epibrassinolide (EBL) (0.1, 0.2, and 0.4 PPM) with or without zinc. Results demonstrated that the application of brassinosteroids, combined with zinc stress, significantly enhanced germination percentage (about 47.06, 63.64, and 120%), speed of germination (about 39.13, 50, and 100%), seedling growth (about 38% in case of treatment combined 0.4 PPM 24-EBL and 1.5 mM ZnSO_4_) and seedling vigor index (204% in case of treatment combined 0.4 PPM 24-EBL and 1.5 mM ZnSO_4_) compared to zinc-treated seedlings alone after 24 h. The activities of antioxidative enzymes (catalase, ascorbate peroxidase, polyphenol oxidase, and peroxidase) and total soluble protein content decreased, while lipid peroxidation and proline content exhibited a significant increase (*p* ≤ 0.05) when compared to the control. However, the negative effects induced by heavy metal stress on these parameters were significantly mitigated by EBL application. Notably, the most effective concentration of EBL in overcoming zinc stress was found to be 0.4 PPM. These findings underscore the potential of exogenously applied brassinosteroids as a valuable tool in phytoremediation projects by ameliorating heavy metal stress.

## 1. Introduction

Contamination of soil and water by heavy metals poses a significant concern and is primarily a result of human activities such as mining, sewage sludge disposal and increased emissions from vehicles and industries (Zorpas et al., 2021). Heavy metals, which generally fall under the category of transition metals, consist of various chemical elements. While some lighter heavy metals like Zn, Co, Cu, Ni, Mn, Mo, and Fe are essential, the majority of them are non-essential. However, all heavy metals can potentially be hazardous depending on their bioavailability levels and the susceptibility of the exposed organism. The detrimental effects of heavy metals extend beyond humans and animals, affecting a wide range of species, including plants. Excessive exposure to heavy metals leads to reduced biomass, leaf chlorosis, delayed root development and morphological abnormalities in plants, ultimately resulting in plant death (Patel et al., 2021). Plants generate reactive oxygen species at different sites within their respiratory and photosynthetic electron transport chains in response to phytotoxic effects. This process induces oxidative stress in cellular systems. Plants have developed a highly efficient antioxidative defense mechanism, encompassing low-molecular-weight antioxidants, to counteract the accumulation of these reactive molecules.

Various approaches, including conventional plant breeding and modern genome editing, have been employed at different times to confer stress resistance in plants. However, due to the advantages and disadvantages associated with these methods, a singular viable solution to address this problem has yet to be discovered. Traditional breeding and marker-assisted selection are cost-effective and widely recognized methods for achieving desirable outcomes (Sharma et al., 2021). Nonetheless, they are time-consuming and face challenges such as reproductive barriers and limited genetic variability. Modern techniques like genetic engineering and genome editing offer potential solutions for stress resistance in plants, but they encounter significant obstacles related to ethical concerns, including germplasm cross-contamination, health risks associated with consumption and public acceptance (Ahmad et al., 2021; Harfouche et al., 2021). In this context, researchers are compelled to explore environmentally friendly techniques that can yield desired results in a shorter timeframe.

Plant growth regulators, specifically brassinosteroids (BRs), naturally occur in trace amounts in all plants and have been extensively studied for their agricultural significance in promoting the growth and yield of various crops. Previous research has also demonstrated the ability of BRs to modulate plant responses to oxidative stress induced by different abiotic stresses (Sharma et al., 2013; Sami et al., 2022; Yaqoob et al., 2022). The potential use of BRs in agriculture lies in their capacity to enhance crop productivity while mitigating abiotic stress. While numerous studies have examined the combined effects of plant hormones on crop performance in normal conditions, limited attention has been paid to the influence of BRs during heavy metal stress.

Mung bean (*Vigna radiata L.*), commonly referred to as green gram, holds a significant position as a traditional crop. It distinguishes itself from other pulses due to its elevated nutritional value. The seeds of *V. radiata* boast a considerable protein content, with dried green gram seeds containing 24.7% protein, 0.6% fat, 0.9% fiber, and 3.7% ash (Maddi and Tayde, 2022). Notably, the mung bean crop forms a symbiotic relationship with specific bacteria, enabling N2 fixation within root nodules to fulfill the plant’s nitrogen requirements. V. radiata finds cultivation across various regions in India, predominantly as a kharif crop. However, in regions with mild winters, like the southern parts, it is grown as a rabi crop. An annual rainfall ranging from 60 to 90 cm proves favorable for this crop. While adaptable to different soil types, mung beans thrive in medium loamy soils. Given the crop’s significance, the current research delves into the interaction between brassinosteroids and zinc, aiming to comprehend their combined impact on seedling growth and the antioxidative defense system during mung bean germination. This study sheds light on the stress-relieving qualities of BRs in this specific context.

## 2. Materials and methods

### 2.1. Chemicals

The chemicals used in this study were procured from M/s Sigma-Aldrich Chemical Co., USA, SRL, Hi-Media Laboratories, Qualigens.

### 2.2. Plant material

Mung bean seeds obtained from the local market were subjected to a washing process using distilled water and then surface sterilized with 0.1% HgCl_2_ for 5 min. Following sterilization, the seeds were rinsed multiple times with sterile distilled water and allowed to imbibe in distilled water for a period of 12 h. For each treatment and control condition, six petri dishes with a diameter of 15 cm were prepared by placing two layers of Whatman No. 1 filter papers. In each Petri dish, ten healthy seeds of similar size were carefully selected and sown.

To create the experimental conditions, different concentrations of 24-EBL alone, ZnSO_4_ alone and a combination of both were prepared and added to the respective Petri plates, with each solution measuring 20 mL (Table 1). The Petri dishes were then placed in a dark room at a temperature of 25 ± 2°C. The different concentrations of 24-EBL (0.1, 0.2 and 0.4 PPM) were labeled as T1, T2 and T3, respectively. The concentrations of ZnSO_4_ (0.5, 1.0 and 1.5 mM) were labeled as T4, T5 and T6, respectively. The combinations of both compounds were labeled as T7 (T1 + T4), T8 (T2 + T5) and T9 (T3 + T6), respectively.

**Table 1:** Details of experimental treatments.

S. No.	Treatments	Descriptions
1	Control	Double distilled Water
2	T1	0.1 PPM 24-EBL
3	T2	0.2 PPM 24-EBL
4	T3	0.4 PPM 24-EBL
5	T4	0.5 mM ZnSO_4_
6	T5	1.0 mM ZnSO_4_
7	T6	1.5 mM ZnSO_4_
8	T7	0.1 PPM 24-EBL + 0.5 mM ZnSO_4_
9	T8	0.2 PPM 24-EBL + 1.0 mM ZnSO_4_
10	T9	0.4 PPM 24-EBL + 1.5 mM ZnSO_4_

### 2.3. Germination and seedling length

The germination percentages and speed of germination were assessed by recording seed germination counts after 24 h (Soares et al., 2020). Radicle development was considered as the indicator of germination. Additionally, the length of seedlings was measured at 24, 72, and 96 h to calculate the seedling vigor index as per the method of Zhao (2016), with the control group serving as the reference for comparison. The following formula was used to calculate the seed germination percentage, speed of germination and seedling vigor index (SVI):

$$\mathrm{Germination}\;\mathrm{percentages}=\frac{\mathrm{No}.\mathrm{of}\;\mathrm{seed}\;\mathrm{germinated}}{\mathrm{Total}\;\mathrm{no}.\mathrm{of}\;\mathrm{seed}}\times 100$$

$$Speed of germination = \sum \frac{Nt}{t\text{.}}$$

Where,

Nt = total number of seed germination in time

t = day of germination


Seedling vigor index = Germination percentage × Seedling length (cm)

### 2.4. Extraction of enzymes

For the analysis of various enzyme activities and other parameters, fresh seedlings were collected at different time points: 2, 4 and 6 days after germination. Three biological replicates and three technical replicates were used. Enzymes such as catalase (CAT) and ascorbate peroxidase (APX) were extracted from germinated seeds (1 g) by homogenizing them in 10 mL of extraction buffer (50 mM potassium phosphate buffer, pH 7.0) using a chilled mortar and pestle. The resulting homogenate was then subjected to centrifugation at 10,000 × *g* for 15 min at 4°C and the supernatant was collected for the determination of CAT and APX activities.

The extraction procedure for peroxidase (POX) and polyphenol oxidase (PPX) enzymes was similar to that used for CAT and APX, with the exception of using a different buffer (Sharma et al., 2013). In this case, a 0.1 M, pH 6.8 buffer was utilized instead of the 50 mM, pH 7.0 buffer used previously.

### 2.5. Quantification of catalase activity

A modified method of the Aebi (1984) was employed to determine the catalase (EC 1.11.1.6) activity. A final reaction volume of 3 mL was achieved by combining 1.4 mL of 50 mM potassium phosphate buffer having pH 7.0, 1.5 mL of 12.5 mM H_2_O_2_ and 0.1 mL of enzyme extract in order to assess CAT activity. By adding H_2_O_2_, the reaction was initiated, while a blank sample was concurrently run without the inclusion of enzyme extract. The decrease in H_2_O_2_ concentration was monitored by measuring the absorbance reduction at 240 nm at 30 s intervals over a 3 min period. This measurement was performed using a double beam UV–VIS spectrophotometer (SPECORD^®^ PLUS 250, Analytik Jena, Germany). One unit (U) of CAT activity was defined as the amount of enzyme catalyzing the decomposition of 1 μmol of H_2_O_2_ per minute at 240 nm, using an extinction coefficient of 0.036 cm^2^ μmol^−1^ for H_2_O_2_ absorbance at 240 nm. The results were expressed as units (U) of CAT activity per gram of fresh weight (U g^−1^ FW).

### 2.6. Quantification of ascorbate peroxidase activity

The activity of ascorbate peroxidase (EC 1.11.1.11) was determined using a modified method based on Kumar et al. (2021) protocol. An assay mixture of 3.0 mL was prepared, which included 1.0 mL of 50 mM potassium phosphate buffer (pH 7.0), 1.0 mL of 39 mM H_2_O_2_, 0.8 mL of 0.5 mM ascorbic acid and 0.2 mL of the enzyme extract. The initiation of the reaction occurred by adding hydrogen peroxide, while a blank sample was concurrently prepared in the absence of the enzyme extract. Over a period of 3 min at 30 s intervals, the rate of ascorbate oxidation at 290 nm was monitored to measure the activity of APX. This measurement was conducted using a double beam UV–VIS spectrophotometer (SPECORD^®^ PLUS 250, Analytik Jena, Germany). One unit of APX activity was defined as the amount required to decompose 1 μmol ascorbic acid oxidized min^−1^ calculated from the extinction coefficient of 2.6 mM^−1^ cm^−1^. The results were expressed as U g^−1^ FW.

### 2.7. Quantification of POX activity

The peroxidase (EC 1.11.1.7) activity was assessed using the method described by Kumar et al. (2021). In this assay, the reaction mixture consisted of o-dianisidine (2.4 μmol), H_2_O_2_ (20 μmol), crude extract (0.05–0.5 mg protein) and 0.05 M citrate buffer (pH 4.8), resulting in a final volume of 3 mL. A blank was prepared by excluding H_2_O_2_ from the incubation mixture. By measuring the absorbance at 430 nm at 15 s intervals, the enzyme activity was determined. The results were reported as U g^−1^ FW.

### 2.8. Quantification of PPO activity

Polyphenol oxidase (EC 1.14.18.1) activity was determined using the method described by Sharma et al. (2013). In this assay, 1 mL of enzyme extract was mixed with 2.5 mL of 0.1 M phosphate buffer (pH 6.8) and 0.1 mL of 0.01 M pyrogallol. Following the preparation of the mixture, it was incubated at 4°C for 5 min. To stop the reaction, 1.0 mL of 2.5 N H_2_SO_4_ was added. In the blank sample, enzyme deactivation was achieved by adding 2.5 N H_2_SO_4_. The measurement of the absorbance at 420 nm was used to determine the amount of purpurogallin formed. One unit (U) of polyphenol oxidase activity corresponds to a 0.01 increase in absorbance at 420 nm per minute caused by the enzyme. The enzyme activity was expressed in U g^−1^ FW.

### 2.9. Estimation of malondialdehyde

Following the method outlined by El-Moshaty et al. (1993), the estimation of malondialdehyde (MDA) was conducted. Tissues weighing two grams from seedlings on the 2nd, 4th and 6th day were homogenized with 3 mL of 0.2 M citrate–phosphate buffer (pH 6.4) in a chilled mortar and pestle. The homogenate was then centrifuged at 10,000 × *g* for 30 min and the resulting supernatant was used for MDA content estimation. To conduct the estimation, 1 mL of the supernatant was combined with an equal volume of MDA reagent, which consisted of 20% trichloroacetic acid in 5% thiobarbituric acid. The mixture was placed in a water bath set at 95°C and incubated for a duration of 40 min. Following the incubation, the mixture was promptly cooled on ice for 15 min. Subsequently, the mixture was subjected to centrifugation at 10,000 × *g* for 30 min. The absorbance of the resulting supernatant was then measured at 520 and 600 nm. The absorbance at 600 nm was subtracted from the absorbance at 520 nm to eliminate non-specific absorbance. Using the extinction coefficient of 155 mM^−1^ cm^−1^, the content of malondialdehyde was calculated. The results were expressed in nanomoles of MDA per gram of fresh weight (nmoles g^−1^ FW).

### 2.10. Estimation of soluble proteins

The estimation of soluble proteins was performed following the method described by Lowry et al. (1951). Fresh leaves weighing one gram were homogenized in 5 mL of phosphate buffer (0.1 M, pH 7.0) using a mortar and pestle. The resulting homogenate was then centrifuged at 10,000 × *g* for 20 min and the supernatant was collected. To 1.0 mL of the extracted solution, 1 mL of 20% trichloroacetic acid (TCA) was added. After allowing it to stand for half an hour, the mixture was centrifuged at 5,000 × *g* for 10 min. The resulting pellet was washed twice with acetone, followed by centrifugation as before and the supernatant was discarded. The pellet was then dissolved in 5 mL of 0.1 N NaOH and used for protein estimation. For protein estimation, 1.0 mL of the protein solution obtained above was mixed with 5 mL of freshly prepared alkaline copper sulfate reagent and thoroughly mixed. A blank was prepared using 1 mL of 0.1 N NaOH in place of the protein sample. After incubating for 10 min at room temperature, 0.5 mL of 1 N Folin-Ciocalteau reagent was added and left in the dark for 30 min. The absorbance of the resulting blue color was measured at 660 nm against the blank. The amount of soluble proteins was calculated in mg g^−1^ FW using a standard plot of bovine serum albumin ranging from 0 to 150 μg.

### 2.11. Estimation of proline content

The estimation of proline content was conducted following the method outlined by Bates et al. (1973). Proline was extracted using sulphosalicylic acid. In the extract, an equal volume of glacial acetic acid and ninhydrin solutions were added. The sample was then heated at 100°C and subsequently cooled. After cooling, 5 mL of toluene was added to the sample. The absorbance of the toluene layer was measured at 528 nm using a spectrophotometer (SPECORD^®^ PLUS 250, Analytik Jena, Germany).

### 2.12. Statistical analysis

Factorial CRD was used to examine the data for the two factors. SAS (Version 9.3, SAS Institute Inc., Cary, NC, USA) was used to compare treatments and number of days for critical difference (CD) at the 5% level of significance. The mean and standard error were calculated and every analysis was done in triplicate.

## 3. Results and discussion

### 3.1. Effect of BR and Zn on germination percentage and speed of germination

**Figure 1:**
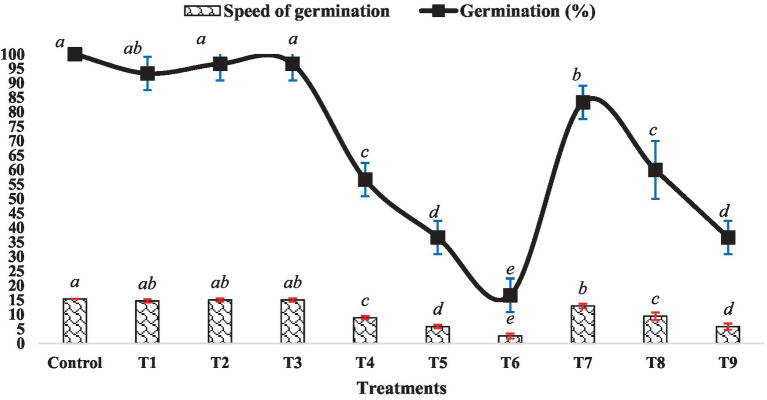
Effect of BR with or without ZnSO_4_ on germination percentage and speed of germination after 24 h of treatment. The shown data are the means of three replications, and the error bars are the standard errors of the means. Alphabets on line and each column indicate the level of significance of at *p* < 0.05. (Where, T1–0.1 PPM 24-EBL; T2–0.2 PPM 24-EBL; T3–0.4 PPM 24-EBL; T4–0.5 mM ZnSO_4_; T5–1.0 mM ZnSO_4_; T6–1.5 mM ZnSO_4_; T7–0.1 PPM 24-EBL + 0.5 mM ZnSO_4_; T8–0.2 PPM 24-EBL + 1.0 mM ZnSO_4_; T9–0.4 PPM 24-EBL + 1.5 mM ZnSO_4_).

The impact of BR and Zn on mung bean’s germination percentage and speed of germination varied across different treatment levels, as depicted in Figure 1. There were no significant differences observed in the germination percentage and speed among treatments T1, T2, T3 and the control group. However, an increase in the level of ZnSO_4_ (from 0.5 mM to 1.5 mM ZnSO_4_) resulted in a decrease in both germination percentage and germination speed. Conversely, treatments T7 to T9 exhibited an increment in both parameters compared to the ZnSO_4_ levels alone. Specifically, treatment T7 showed a 39.13% increase in germination speed and a 47.06% increase in germination percentage compared to treatment T4. Similarly, treatment T8 displayed increments of approximately 50 and 63.64%, respectively as compared to treatment T5. Treatment T9 exhibited the maximum improvement, with a 100% increase in germination speed and a 120% increase in germination percentage compared to treatment T6. The outcomes highlight the intricate interplay between BR and Zn concentrations in influencing mung bean germination. While ZnSO_4_ at higher concentrations appeared to suppress germination, the inclusion of BR counteracted these inhibitory effects, leading to improved germination attributes. This suggests a potential synergistic effect between BR and lower Zn concentrations. Numerous studies have demonstrated that BR treatment significantly (*p* < 0.05) enhances seed germination percentage and germination speed. An instance of this can be seen in the study conducted by Basit et al. (2021), where they discovered that EBL treatment had a significant positive impact on the germination characteristics of rice. Likewise, Basit et al. (2022b) observed improved seed germination percentage and other related parameters in soybean seeds treated with EBL when exposed to chromium toxicity.

### 3.2. Effect of BR and Zn on seedling growth and seedling vigor index

The effect of BR and Zn on seedling growth and seedling vigor index (SVI) is presented in Table 2. A significant reduction in seedling length and SVI was observed with an increase in heavy metal concentration (from 0.5 mM to 1.5 mM ZnSO_4_) compared to the control condition after 24 h. Similar trends were observed for seedling length and SVI after 72 and 96 h. These results strongly suggest that elevated ZnSO_4_ concentrations have a substantial inhibitory effect on seedling growth and vigor, potentially hindering the initial stages of seedling development. In contrast, individual BR treatments (T1 to T3) did not yield statistically significant differences in terms of seedling length and SVI after 24, 72, and 96 h. This suggests that, within the scope of this study, BR application alone did not lead to observable effects on seedling growth and vigor. This could imply that BR alone might not be a sole determinant of seedling development and that its influence might be subject to interactions with other factors. Conversely, the results take an intriguing turn when considering combined BR and Zn treatments (T7 to T9). These combined treatments showcased a clear positive impact, leading to enhanced seedling length and SVI compared to treatments with Zn alone (T4 to T6) across all time intervals. This phenomenon suggests a potential synergistic effect between BR and Zn in promoting seedling growth and vigor. This observation aligns with the complex interplay of plant hormones and essential nutrients in influencing plant physiology. The maximum improvement was observed in treatment T9, with approximately 38% increase in seedling length and approximately 204% increase in SVI compared to treatment T6. These findings align with the results reported by Basit et al. (2022a), who observed that rice under chromium stress exhibited decreased seedling length and vigor index, but supplementation with BR externally enhanced both parameters. Similarly, Srivastava et al. (2011) demonstrated that the application of BR can stimulate seed germination parameters in mung bean.

**Table 2:** Effect of BR with or without ZnSO4 on seedling length and seedling vigor index after 24, 72, and 96 h.

	Seedling length (cm)			Seedling vigor index (SVI)		
	24 h	72 h	96 h	24 h	72 h	96 h
Control	4.1 ± 0.16*^cd^*	8.7 ± 0.32*^cd^*	12.5 ± 0.35*^cde^*	396 ± 10.30*^ab^*	870 ± 32.47*^bc^*	1,250 ± 34.70*^bc^*
T1	4.6 ± 0.20*^abc^*	9.2 ± 0.17*^bc^*	12.9 ± 0.44*^bc^*	446 ± 44.06*^ab^*	920 ± 16.59*^ab^*	1,290 ± 44.19*^ab^*
T2	4.9 ± 0.10*^ab^*	9.6 ± 0.19*^ab^*	13.4 ± 0.40*^ab^*	490 ± 9.72*^ab^*	960 ± 19.04*^ab^*	1,340 ± 39.86*^ab^*
T3	5.1 ± 0.20 *^a^*	10.2 ± 0.30*^a^*	14.0 ± 0.24*^a^*	510 ± 20.01*^a^*	1,020 ± 30.01*^a^*	1,400 ± 23.98*^a^*
T4	3.8 ± 0.06*^de^*	7.4 ± 0.18*^e^*	11.9 ± 0.53*^e^*	215 ± 18.98*^de^*	447 ± 10.97*^de^*	714 ± 31.54*^d^*
T5	3.3 ± 0.09*^ef^*	6.4 ± 0.17*^f^*	11.1 ± 0.12*^f^*	121 ± 16.32*^ef^*	256 ± 6.92*^f^*	444 ± 4.80*^e^*
T6	2.9 ± 0.07*^f^*	5.9 ± 0.20*^f^*	10.4 ± 0.36*^g^*	48 ± 16.09*^f^*	98 ± 31.74*^g^*	175 ± 64.64*^f^*
T7	4.6 ± 0.09*^abc^*	9.0 ± 0.18*^bc^*	13.0 ± 0.12*^bc^*	383 ± 19.07*^bc^*	780 ± 50.84*^c^*	1,127 ± 82.42*^c^*
T8	4.3 ± 0.07*^bcd^*	8.6 ± 0.38*^cd^*	12.6 ± 0.35*^cd^*	258 ± 39.04*^cd^*	545 ± 59.96*^d^*	798 ± 81.02*^d^*
T9	4.0 ± 0.15*^cd^*	8.2 ± 0.27*^d^*	12.1 ± 0.26*^de^*	146 ± 19.97*^def^*	327 ± 74.03*^ef^*	485 ± 127.34*^e^*

Values represent the mean ± SE. Alphabets in superscript indicate the level of significance of at *p* < 0.05; Where, T1–0.1 PPM 24-EBL; T2–0.2 PPM 24-EBL; T3–0.4 PPM 24-EBL; T4–0.5 mM ZnSO4; T5–1.0 mM ZnSO4; T6–1.5 mM ZnSO4; T7–0.1 PPM 24-EBL + 0.5 mM ZnSO4; T8–0.2 PPM 24-EBL + 1.0 mM ZnSO4; T9–0.4 PPM 24-EBL + 1.5 mM ZnSO4.

### 3.3. Effect of BR and Zn on CAT activity

**Figure 2:**
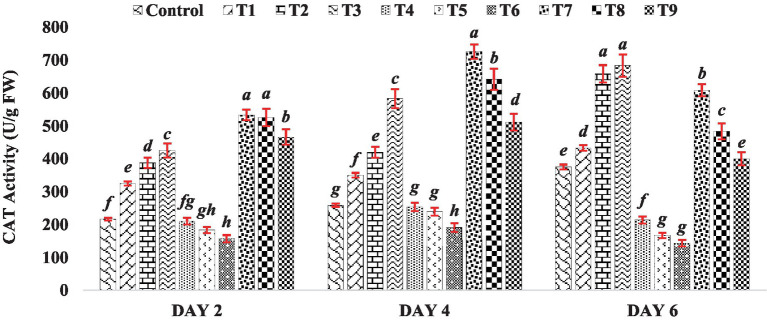
Effect of BR with or without ZnSo_4_ on catalase activity. The shown data are the means of three replications, and the error bars are the standard errors of the means. Alphabets in each column indicate the level of significance of at *p* < 0.05. (Where, T1–0.1 PPM 24-EBL; T2–0.2 PPM 24-EBL; T3–0.4 PPM 24-EBL; T4–0.5 mM ZnSO_4_; T5–1.0 mM ZnSO_4_; T6–1.5 mM ZnSO_4_; T7–0.1 PPM 24-EBL + 0.5 mM ZnSO_4_; T8–0.2 PPM 24-EBL + 1.0 mM ZnSO_4_; T9–0.4 PPM 24-EBL + 1.5 mM ZnSO_4_).

The catalase activity in seedlings of mung bean seeds treated with BR was consistently higher compared to the control group. Specifically, among all the treatments of BR alone (T1 to T3), mung bean seedlings exhibited the highest catalase activity at a higher concentration of BR (Treatment T3), which was at 0.4 PPM, across all stages of seedling growth. This trend underscores the concentration-dependent impact of BR on catalase activity, highlighting its potential role as an inducer of antioxidative responses. When mung bean seeds were exposed to varying concentrations of zinc sulfate (Treatments T4 to T6), their catalase activity in seedlings decreased. The reduction in catalase activity suggests a potential interference of ZnSO_4_ with the normal functioning of antioxidative pathways, which could be attributed to the intricate interactions between heavy metals and plant defense mechanisms. However, when different concentrations of BR were combined with zinc (Treatments T7 to T9), the catalase activity was higher than that observed when zinc sulfate was present alone. This observation is depicted in Figure 2, where the catalase activity in the treatments with both BR and zinc was higher compared to the treatments with zinc sulfate alone. The results indicate that the treatment with BR stimulated catalase activity, leading to enhanced antioxidative metabolism in the germinated mung bean seedlings. On the other hand, the reduction in CAT activity observed in the presence of zinc sulfate alone suggests that zinc sulfate suppresses the antioxidative metabolism of the seedlings. The findings from previous research studies conducted on other crops, such as those by Arora et al. (2010) and Tadaiesky et al. (2021), have shown a similar effect of brassinosteroids. These studies observed that the application of brassinosteroids resulted in increased CAT activity. In the current study, when BR was applied in combination with zinc sulfate, the catalase activity was higher compared to the presence of zinc alone. This suggests that BR had a mitigating effect on the adverse impact of zinc, further supporting the notion that BR can alleviate the negative effects of zinc on antioxidative metabolism.

### 3.4. Effect of BR and Zn on APX activity

**Figure 3:**
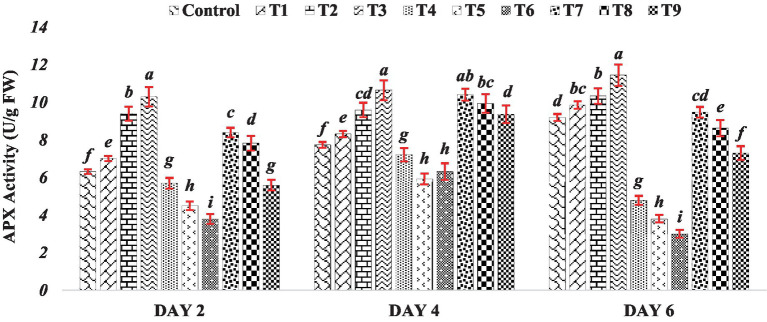
Effect of BR with or without ZnSO_4_ on APX activity. The shown data are the means of three replications, and the error bars are the standard errors of the means. Alphabets in each column indicate the level of significance of at *p* < 0.05. (Where, T1–0.1 PPM 24-EBL; T2–0.2 PPM 24-EBL; T3–0.4 PPM 24-EBL; T4–0.5 mM ZnSO_4_; T5–1.0 mM ZnSO_4_; T6–1.5 mM ZnSO_4_; T7–0.1 PPM 24-EBL + 0.5 mM ZnSO_4_; T8–0.2 PPM 24-EBL + 1.0 mM ZnSO_4_; T9–0.4 PPM 24-EBL + 1.5 mM ZnSO_4_).

The activity of ascorbate peroxidase gradually increased under the influence of BR alone (Treatments T1 to T3). This observed trend suggests that BR application might serve as a positive modulator of APX activity, potentially enhancing the antioxidative capacity of mung bean seedlings. This aligns with the established role of BR in promoting various stress responses, including antioxidative defenses, in plants (Kaur et al., 2022; Manghwar et al., 2022). In stark contrast, the response to varying concentrations of zinc sulfate (Treatments T4 to T6) presents an opposing trend. The APX activity decreased as the concentration of zinc sulfate increased. This observation indicates that higher concentrations of zinc sulfate could potentially inhibit the normal functioning of APX, thereby diminishing the seedlings’ antioxidative capabilities. The inverse relationship between zinc concentration and APX activity suggests a potential vulnerability of antioxidative pathways to the inhibitory effects of elevated zinc levels. This response was consistent across Day 2, Day 4 and Day 6. The data presented in Figure 3 demonstrates that the decline in APX activity was more pronounced with higher concentrations of zinc sulfate. However, when the combination of BR and zinc was used (Treatments T7 to T9), APX exhibited higher activity compared to the activity observed in the presence of zinc sulfate alone (Treatments T4 to T6). The observed results suggest that the application of both BR and zinc sulfate had a relieving effect on seedlings subjected to zinc metal stress by influencing antioxidative metabolism. The activity of APX is crucial in detoxifying ROS and mitigating oxidative stress. APX plays a significant role in the reduction of hydrogen peroxide to water through the ascorbate-glutathione cycle (Hasanuzzaman et al., 2019). Therefore, the higher APX activity observed in the treatments with both BR and zinc sulfate indicates that they played a role in ameliorating oxidative stress and reducing the detrimental effects of zinc metal stress on the seedlings.

### 3.5. Effect of BR and Zn on POX

**Figure 4:**
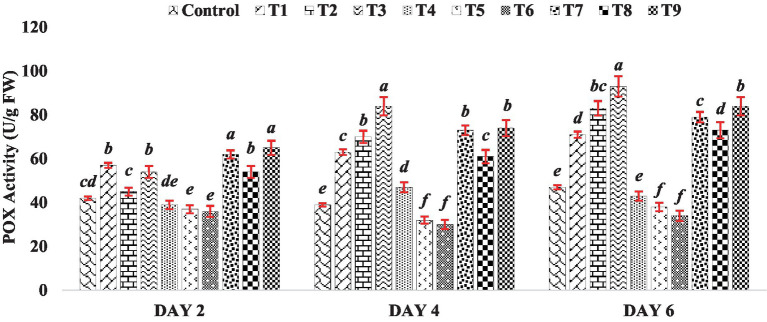
Effect of BR with or without ZnSO_4_ on POX activity. The shown data are the means of three replications, and the error bars are the standard errors of the means. Alphabets in each column indicate the level of significance of at *p* < 0.05. (Where, T1–0.1 PPM 24-EBL; T2–0.2 PPM 24-EBL; T3–0.4 PPM 24-EBL; T4–0.5 mM ZnSO_4_; T5–1.0 mM ZnSO_4_; T6–1.5 mM ZnSO_4_; T7–0.1 PPM 24-EBL + 0.5 mM ZnSO_4_; T8–0.2 PPM 24-EBL + 1.0 mM ZnSO_4_; T9–0.4 PPM 24-EBL + 1.5 mM ZnSO_4_).

The peroxidase activity in the mung bean increased notably compared to the control when exposed to different concentrations of BR treatments (T1 to T3), except for treatment T2 on Day 2 (Figure 4). Conversely, when different concentrations of zinc were used alone (Treatments T4 to T6), zinc exhibited an inhibitory effect on POX activity, as depicted in Figure 4. However, when BR was applied in combination with zinc (Treatments T7 to T9), the activity of POX was enhanced compared to the presence of zinc alone. This indicates the stress-alleviating effect of BR. Similar to CAT, POX also plays a role in breaking down H_2_O_2_ into water and oxygen. The results of POX activity in mung bean demonstrated an increase in enzyme activity after the application of BR, either alone or in combination with zinc, at different concentrations. Indeed, other researchers, such as Arora et al. (2010) and Soares et al. (2020), have also reported an increase in POX activity in response to the application of brassinosteroids. POX is involved in the synthesis of lignin and other phenolic polymers. The enhancement in POX activity may serve as a defense mechanism for cells against harmful concentrations of hydroperoxides, thereby protecting cellular components such as proteins and lipids from oxidation. This notion aligns with the findings of Beauchamp and Fridovich (1971), who suggested that increased POX activity helps safeguard cellular components against oxidation.

### 3.6. Effect of BR and Zn on PPO activity

**Figure 5:**
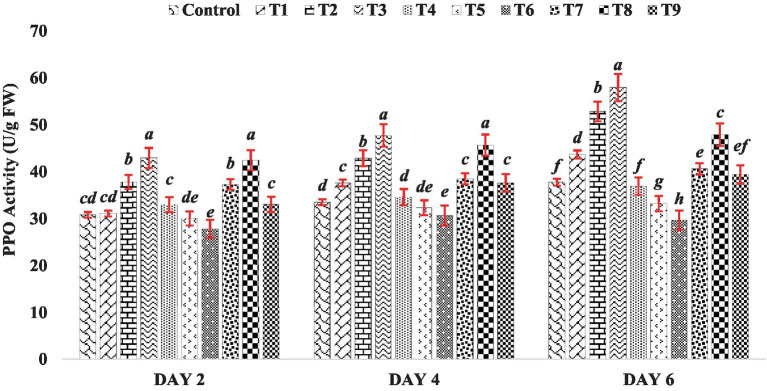
Effect of BR with or without ZnSO_4_ on PPO activity. The shown data are the means of three replications, and the error bars are the standard errors of the means. Alphabets in each column indicate the level of significance of at *p* < 0.05. (Where, T1–0.1 PPM 24-EBL; T2–0.2 PPM 24-EBL; T3–0.4 PPM 24-EBL; T4–0.5 mM ZnSO_4_; T5–1.0 mM ZnSO_4_; T6–1.5 mM ZnSO_4_; T7–0.1 PPM 24-EBL + 0.5 mM ZnSO_4_; T8–0.2 PPM 24-EBL + 1.0 mM ZnSO_4_; T9–0.4 PPM 24-EBL + 1.5 mM ZnSO_4_).

In this experiment, the activity of PPO increased as the concentration of BR increased (Treatments T1 to T3) on day 2, 4 and 6 of seedling growth (Figure 5). Among all the BR treatments, the highest PPO activity was observed at a concentration of 0.4 PPM (Treatment T3). However, when mung bean seeds were treated with zinc alone (Treatments T4 to T6), the seedlings showed an inhibitory effect on PPO activity throughout the sampling (Day 2, 4 and 6), as shown in Figure 5. Interestingly, when mung bean seeds were treated with both BR and zinc sulfate (Treatments T7 to T9), the PPO activity was substantially higher compared to the presence of zinc alone (Treatments T4 to T6). This suggests that BR plays a protective role against heavy metal stress, as it enhances PPO activity and counteracts the inhibitory effect of zinc on PPO activity, indicating a potential mechanism for mitigating the negative effects of heavy metals on mung bean seedlings. The results of this experiment indicate that both BR alone and in combination with different concentrations of zinc metal were effective in enhancing the activity of polyphenol oxidase compared to the control group. This finding is consistent with the observations made by Asghari and Rezaei-Rad (2018), who reported an increase in PPO activity in table grape when treated with BRs. These findings suggest that BRs have a positive impact on PPO activity and may contribute to the improvement of physiological processes related to polyphenol metabolism in plants.

### 3.7. Effect of BR and Zn on lipid peroxidation

Oxidative stress can lead to lipid peroxidation of the cell membrane, resulting in the production of malondialdehyde. However, in this experiment, it was observed that the content of MDA reduced in germinated mung beans treated with BR (Table 3). The decline in MDA content was more pronounced when BR was applied in higher concentrations (Treatments T1 to T3). This decline in MDA level indicated that BR treatment caused a decrease in lipid peroxidation, implying that BR has antioxidative properties. Conversely, treatment with varying concentrations of zinc metal alone (Treatments T4 to T6) caused an increase in MDA content, indicating an increase in lipid peroxidation. The highest MDA content was observed in treatment T6, which suggests that higher concentrations of zinc induced greater lipid peroxidation. When mung bean seeds were treated with a combination of Zn and BR (Treatments T7 to T9), the content of MDA initially decreased. However, with an increase in the concentration of both Zn and BR, the MDA content increased. This indicates that the combined treatment initially mitigated lipid peroxidation, but at higher concentrations of Zn and BR, there might have been a reversal, resulting in an increase in lipid peroxidation. In summary, the results suggest that BR treatment reduced lipid peroxidation (as evident from the decline in MDA content), while zinc treatment increased lipid peroxidation. The combined treatment of Zn and BR showed a complex effect on MDA content, with an initial decrease followed by a subsequent increase at higher concentrations of both Zn and BR. Previous research by Arora et al. (2010) supports the observation that the level of MDA decreases with an increase in the concentration of BRs in the presence of zinc stress in *Zea mays.*

**Table 3:** Effect of BR with or without ZnSO_4_ on MDA, total soluble protein and proline content.

	MDA content (nmoles/g FW)			Total soluble protein (mg/g FW)			Proline content (μg/g FW)		
	Day 2	Day 4	Day 6	Day 2	Day 4	Day 6	Day 2	Day 4	Day 6
Control	13.10 ± 0.07*^de^*	17.40 ± 0.16*^ab^*	15.70 ± 0.05*^b^*	45.79 ± 0.42*^fg^*	76.83 ± 2.69*^de^*	88.67 ± 1.55*^de^*	5.12 ± 0.22*^gh^*	6.22 ± 0.07*^f^*	7.35 ± 0.22*^g^*
T1	12.87 ± 0.24*^e^*	16.80 ± 0.34*^bc^*	15.40 ± 0.33*^b^*	68.00 ± 1.15*^c^*	83.97 ± 1.05*^c^*	94.51 ± 1.61*^c^*	4.64 ± 0.17*^h^*	6.73 ± 0.13*^ef^*	7.87 ± 0.03*^fg^*
T2	11.21 ± 0.56*^f^*	15.60 ± 0.43*^de^*	15.20 ± 0.63*^b^*	79.56 ± 1.50*^b^*	98.55 ± 0.27*^b^*	107.99 ± 3.47*^b^*	5.53 ± 0.04*^g^*	7.60 ± 0.21*^d^*	8.71 ± 0.13*^e^*
T3	9.83 ± 0.43*^g^*	14.80 ± 0.37*^e^*	15.10 ± 0.54*^b^*	93.77 ± 3.02*^a^*	114.78 ± 2.47*^a^*	134.97 ± 3.14*^a^*	6.79 ± 0.28*^ef^*	8.85 ± 0.36*^bc^*	9.94 ± 0.04*^bcd^*
T4	14.40 ± 0.72*^bc^*	17.10 ± 0.36^bc^	14.60 ± 0.07*^b^*	53.54 ± 1.29*^de^*	81.61 ± 0.73*^cd^*	93.84 ± 2.43*^cd^*	6.21 ± 0.26*^f^*	7.33 ± 0.21*^de^*	8.50 ± 0.33*^ef^*
T5	15.20 ± 0.09*^ab^*	17.25 ± 0.40*^abc^*	15.30 ± 0.44*^b^*	49.75 ± 0.93*^ef^*	72.58 ± 1.75*^e^*	83.80 ± 2.24*^ef^*	7.43 ± 0.29*^de^*	8.58 ± 0.29*^c^*	9.75 ± 0.04*^cd^*
T6	16.30 ± 0.10*^a^*	18.24 ± 0.13*^a^*	16.90 ± 0.72*^a^*	42.70 ± 0.72*^g^*	66.51 ± 0.24*^f^*	78.52 ± 1.28*^fg^*	8.11 ± 0.20*^bc^*	9.29 ± 0.39*^b^*	10.50 ± 0.28*^b^*
T7	14.60 ± 0.29*^bc^*	16.90 ± 0.58*^bc^*	15.40 ± 0.43*^b^*	58.81 ± 2.04*^d^*	72.63 ± 2.14*^e^*	77.48 ± 1.93*^g^*	7.56 ± 0.16*^cd^*	8.67 ± 0.03*^bc^*	9.54 ± 0.35*^d^*
T8	14.00 ± 0.16*^cd^*	16.20 ± 0.61*^cd^*	15.00 ± 0.58*^b^*	67.78 ± 0.85*^c^*	86.71 ± 3.80*^c^*	95.77 ± 3.85*^c^*	8.26 ± 0.22*^b^*	9.24 ± 0.02*^bc^*	10.39 ± 0.37*^bc^*
T9	14.40 ± 0.50*^bc^*	16.80 ± 0.03*^bc^*	15.30 ± 0.30*^b^*	72.92 ± 1.30*^c^*	97.59 ± 1.84*^b^*	130.63 ± 2.94*^a^*	8.99 ± 0.31*^a^*	10.12 ± 0.39*^a^*	11.28 ± 0.39*^a^*

Values represent the mean ± SE. Alphabets in superscript indicate the level of significance of at *p* < 0.05; Where, T1–0.1 PPM 24-EBL; T2–0.2 PPM 24-EBL; T3–0.4 PPM 24-EBL; T4–0.5 mM ZnSO4; T5–1.0 mM ZnSO4; T6–1.5 mM ZnSO4; T7–0.1 PPM 24-EBL + 0.5 mM ZnSO4; T8–0.2 PPM 24-EBL + 1.0 mM ZnSO4; T9–0.4 PPM 24-EBL + 1.5 mM ZnSO4.

This decrease in MDA content can be attributed to the enhanced activities of antioxidative enzymes. This suggests that BRs may serve as secondary messengers, triggering the induction of the antioxidant defense system in stressed plants. Consequently, BRs effectively scavenge reactive oxygen species in plants experiencing stress. This aligns with the proposed role of BRs in activating the antioxidant defense system and mitigating oxidative stress in plants under challenging conditions, as suggested by Hu et al. (2021) and Kretynin et al. (2021).

### 3.8. Effect of BR and Zn on total soluble proteins content

The level of total soluble proteins in mung bean seedlings was influenced by the application of BRs (Table 3). It was observed that there was an increase in the soluble protein content, indicating that the BR responses were dependent on protein synthesis. This finding is in line with the suggestion made by Siddiqi and Husen (2021), who proposed that BRs can modulate protein synthesis and contribute to changes in the level of soluble proteins. The increase in soluble protein content suggests that BRs may play a role in regulating protein metabolism and synthesis in mung bean seedlings, potentially contributing to the overall response to BR treatment. In the presence of zinc stress alone (Treatments T4 to T6), the content of soluble protein in germinating mung seedlings decreased with increasing concentrations of zinc metal. However, when BR concentrations were applied together with the zinc concentrations (Treatments T7 to T9), there was a gradual increase in the content of soluble protein. These observations suggest that BR alleviated the zinc stress in germinating mung seeds. It can be inferred from these findings that BRs have the ability to enhance protein content even under normal conditions in plants, as reported by Arora et al. (2010) and Sharma et al. (2013).

### 3.9. Effect of BR and Zn on proline content

In this experiment, it was observed that mung bean seedlings accumulated proline content in response to zinc stress and the supplementation of BRs further increased the proline content (Table 3). Proline is known to accumulate in plants as a response to abiotic stresses, including zinc stress. The accumulation of proline serves as a protective mechanism for plants under stress conditions. Proline acts as a compatible solute, helping to maintain osmotic balance and stabilizing cellular structures and enzymes. It plays a crucial role in alleviating stress on enzymes and cellular structures by acting as a scavenger of reactive oxygen species and protecting macromolecules from oxidative damage. The higher proline content observed in mung bean seedlings treated with BRs suggests that BRs enhance the plant’s ability to cope with zinc stress by promoting proline accumulation. Indeed, the role of proline as an organic nitrogen store during recovery from stress has been proposed (El Moukhtari et al., 2020). In the context of BRs, their induction of aluminum stress tolerance in mung bean was associated with increased levels of free proline (Ali et al., 2008). Additionally, Li et al. (2022) demonstrated that brassinosteroid-induced cadmium stress tolerance in grape seedlings was accompanied by stimulated proline accumulation. These studies further support the notion that proline accumulation is a common response mechanism induced by BRs under various stress conditions, including zinc stress in mung bean seedlings. The enhanced proline levels observed in the present study suggest that BRs play a crucial role in promoting stress tolerance by modulating proline metabolism.

## 4. Conclusion

To sum up, the utilization of BR treatments holds promise in enhancing seed germination, seedling growth and other biochemical activities in mung beans under heavy metal stress. The positive effects observed on seed germination and seedling growth following BR treatments are likely attributed to an increase in total soluble protein and proline content. The application of brassinosteroids further augmented the activity of antioxidative enzymes in response to Zn stress. Consequently, it is plausible that the reinforced antioxidant system played a significant role in conferring resistance to Zn stress in mung bean seedlings. These findings suggest that employing BRs as a novel approach could enhance seedling growth in the presence of Zn stress and prove beneficial for phytoremediation initiatives.

## Data availability statement

The original contributions presented in the study are included in the article/supplementary material, further inquiries can be directed to the corresponding authors.

## Author contributions

NK: Data curation, Investigation, Writing – original draft. VS: Data curation, Investigation, Writing – original draft. GK: Data curation, Investigation, Writing – original draft. CL: Formal analysis, Writing – review & editing. HD: Writing – review & editing, Formal analysis. KP: Writing – review & editing. FK: Writing – review & editing. VG: Writing – review & editing. MK: Writing – review & editing.
